# Prediction of chromatin looping using deep hybrid learning (DHL)

**DOI:** 10.15302/J-QB-022-0315

**Published:** 2023-06-01

**Authors:** Mateusz Chiliński, Anup Kumar Halder, Dariusz Plewczynski

**Affiliations:** ^1^ Faculty of Mathematics and Information Sciences Warsaw University of Technology 00‐662 Warsaw Poland; ^2^ Laboratory of Functional and Structural Genomics Centre of New Technologies University of Warsaw 02‐097 Warsaw Poland

**Keywords:** deep learning, 3D genomics, transformers, spatial organisation of nucleus, ChIA‐PET, DNA‐Seq

## Abstract

**Background:**

With the development of rapid and cheap sequencing techniques, the cost of whole‐genome sequencing (WGS) has dropped significantly. However, the complexity of the human genome is not limited to the pure sequence—and additional experiments are required to learn the human genome’s influence on complex traits. One of the most exciting aspects for scientists nowadays is the spatial organisation of the genome, which can be discovered using spatial experiments ( ***e.g.***, Hi‐C, ChIA‐PET). The information about the spatial contacts helps in the analysis and brings new insights into our understanding of the disease developments.

**Methods:**

We have used an ensemble of deep learning with classical machine learning algorithms. The deep learning network we used was DNABERT, which utilises the BERT language model (based on transformers) for the genomic function. The classical machine learning models included support vector machines (SVMs), random forests (RFs), and K‐nearest neighbor (KNN). The whole approach was wrapped together as deep hybrid learning (DHL).

**Results:**

We found that the DNABERT can be used to predict the ChIA‐PET experiments with high precision. Additionally, the DHL approach has increased the metrics on CTCF and RNAPII sets.

**Conclusions:**

DHL approach should be taken into consideration for the models utilising the power of deep learning. While straightforward in the concept, it can improve the results significantly.

## Abstract 2

**Author summary:** Deep neural networks have revolutionised every aspect of life. In our work, we are applying them along with other classical machine learning algorithms to create a robust artificial intelligence algorithm that can predict interactions in the nucleus. For that task, only pure DNA sequence is used, which can be obtained using well‐known techniques, and is not only very cheap but also broadly available, both in scientific laboratories and with the help of commercially available kits.

## INTRODUCTION

The function of the genome has been interesting for scientists for decades, yet there is still a lot to discover. Multiple significant steps have already been done—from creating the first assembly of the human genome [1,2] through numerous population [3, 4, 5] and genome‐wide association studies (GWAS) studies [6]. However, as the field was developing and cheap and rapid sequencing technologies were introduced, it turned out that GWAS studies could not explain all the complex diseases and heritability. Multiple other experiments were proposed to explain the unknown parts, including ATAC‐Seqs, ChIP‐Seqs, and the whole range of spatial experiments. The last ones are essential—they bring together far‐apart genome regions and allow the genome to regulate itself. Studying the spatial landscape of the genome allows going beyond the linear experiments, and brings new insights into the analysis. Those contacts are not rare—the 1.8 meters long DNA strain must be packed tightly into a sphere‐shaped nucleus with a diameter of 6–10 μm [7].

That is why multiple methods of capturing the spatial landscape were introduced—starting with all the 3C methods [8] (that allowed testing of the spatial closeness in one vs. one fashion), introduced in 2002, developing through 4C methods [9] (allowing one vs. all comparisons), up to currently more popular methods—Hi‐C [10] and ChIA‐PET (chromatin interaction analysis by paired‐end tag sequencing) [11], which capture the interactions occurring in the genome using all vs. all approach. However, those experiments are very time‐ and cost‐consuming. The advancement of artificial intelligence in the field of computational biology raise the questions again—if the genome is creating those interactions, should it not be encoded in the sequence as well? That question fueled the search for the connection between the linear, 1D sequence and the spatial interactions. The easy connection has not been established yet; however, multiple tries have been made, especially for predicting Hi‐C interactions using linear sequence, with the help of deep learning [12,13].

With the advance in the field, multiple algorithms have been created to decipher the language of non‐coding regions of DNA. Deep learning‐based methods are successfully applied in biological sciences, especially with the genomic sequence, to understand each aspect of cis‐regulatory elements and their mechanism ( *e.g.*, identification of non‐coding variants, protein‐DNA interaction, and chromatin accessibility). Among the class of deep learning algorithms, bidirectional encoder representations from transformers (BERT) [14] is advantageous for understanding DNA sequence data compared to the other convolutional neural network (CNN) based architectures [15]. BERT as a language model can capture contextual information from the sequence data with attention mechanisms and develop a general understanding of the complete system. More specifically, this architecture has the capability to transfer this understanding into various tasks related to the learned biological system, in contrast to recurrent neural networks (RNNs) [16] and CNNs, which fail to satisfy these requirements.

To show the power of BERT models in biology, DNABERT [17] was created—a pre‐trained BERT model introduced to explore human DNA as a language model. The fine‐tuning stage of the DNABERT is aimed at several biological prediction tasks, such as finding the proximal and core promoter regions and transcription factor binding sites.

Inspired by the idea of DNABERT and its general‐purpose pre‐training model, we propose a deep hybrid learning (DHL) system for 3D genome‐wide chromatin interaction prediction from linear DNA sequence. We incorporate the DNABERT pre‐trained model in the proposed scheme and devise a fine‐tuning method for chromatin loop prediction. In the next step, we introduce classical machine learning algorithms, support vector machine (SVM) [18], random forest (RF) [19], and K‐nearest neighbor (KNN) [20], and devise the final hybrid learning system.

## RESULTS

The proposed DHL‐based approach predicts genome‐wide high‐resolution 3D chromatin interactions mediated by CTCF and RNAPII using 1D genomic sequence information. The training and testing data set in this experiment are constructed at the whole genome level, with the restriction of chromatin interactions smaller than 1 Mb. The set of interactions for the testing set is created from chromosome 9 of the GM12878 cell line, while all the remaining chromosomes are used for training. The training and validation for all the classifiers are evaluated with statistical metrics: Accuracy (ACC), Precision (Prec), Recall, F1 measure, Matthew’s correlation coefficient (MCC), and Area under the ROC Curve (AUC).

**Figure 1:**
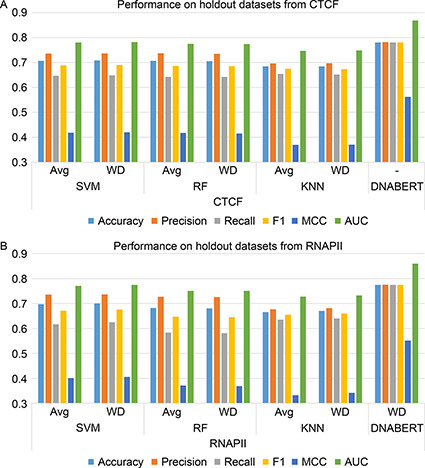
The performance evaluation on the holdout datasets from (A) CTCF and (B) RNAPII.

In the first step of our approach, all the individual models are trained using the beforementioned classical machine learning algorithms (MLAs) and DNABERT. The metrics of MLAs are presented in two ways: fold‐wise (incorporated for cross‐validation (CV)) and WD‐based (that provides equity in learning with respect to the DNABERT approach). The performance scores for CTCF and RNAPII datasets are reported in the Fig.1. The scores from CV models are represented as the average (Avg) of all fold‐wise performances. More detailed training metrics are reported in the Material and Methods.

Further, a pre‐trained DNABERT model is used; however, it requires task‐specific fine‐tuning for the chromatin loop prediction. After fine‐tuning, DNABERT model achieves a better accuracy score in comparison to MLAs (5‐fold cross‐validated)‐0.7808 (CTCF) and 0.775 (RNAPII) compared to the individual classical machine learning algorithms, SVM (ACC‐ Avg: 0.707, and WD: 0.708 in CTCF, and Avg: 0.697, and WD: 0.700 in RNAPII), RF (ACC‐Avg: 0.706, WD: 0.705 in CTCF and Avg: 0.682, WD: 0.680 in RNAPII), KNN (ACC‐Avg: 0.684, WD: 0.684 in CTCF and Avg: 0.665, WD: 0.670 in RNAPII). The detailed performance scores are presented in the Fig.1.

The performance scores are consistent among the testing set, as shown in the Fig.1. Within individual‐level classification, DNABERT obtains the highest performance for all evaluation metrics. In the first phase of the hybrid model, the predictions of all three machine learning algorithms are combined into a single decision introducing a logical operation over the results. In the next phase, those decisions are integrated with DNABERT results to obtain the final DHL prediction. The accuracy has been significantly improved in DHL by ~18.22% and 13.28% compared to MLA and ~7.69% and ~9.006% compared to DNABERT for CTCF and RNAPII datasets, respectively. The metrics of the DHL approach are reported in the Tab.1.

To make our tool comparable with other deep learning predictors (however, not entirely, as the other predictors predict the strength of the contacts, which is in the form of regression, while we are interested in strictly binary looping), we have additionally processed loops obtained from the Hi‐C data from Rao *et al*., 2014 [21]. We have taken the loops that were generated by the authors, and used our processing pipeline to convert them into training, and testing set (with the whole pipeline exactly the same as described in the Material and Methods). We have obtained accuracy around 60%, which is quite good, considering that the resolution of the loops was 5 kb, and DNA‐BERT is limited by 256 bp in each anchor. We strongly believe, that with the increase of that limit, the accuracy would go up.

**Table 1:** The performance evaluation of the DHL model for both datasets CTCF and RNAPII

Dataset	Classifier	Accuracy	Precision	Recall	F1	MCC
CTCF	DNABERT	0.7808	0.782	0.7808	0.7806	0.562
	ALL‐ML	0.7113	0.7425	0.6468	0.6914	0.4261
	DHL	0.8409	0.8566	0.819	0.8374	0.6826
RNAPII	DNABERT	0.775	0.776	0.775	0.775	0.552
	ALL‐ML	0.7457	0.7959	0.6608	0.7221	0.4986
	DHL	0.8448	0.8675	0.8139	0.8398	0.6909

## DISCUSSIONS

The *in‐silico* predictions become crucial to understanding the mechanisms of transcriptional regulation from the perspective of chromatin looping. This work presents a deep hybrid learning strategy by including a pre‐trained BERT architecture [14] and three classical machine learning algorithms to predict the 3D chromatin loops from its sequence information. The results described above show that the proposed DHL is stable and robust. It will provide a convenient and effective way to improve our knowledge about the gene regulation effect of chromatin interactions. With the advance of technology, laboratories and companies all over the world are sequencing millions of genomes. However, not everything can be easily explained with just the genetic variation—in some cases even huge GWAS cannot answer the questions that we have, especially about the complex diseases origin and causes. Thus, scientistcs need to use additional experiments, including ChIP‐Seqs, ATAC‐Seqs, Hi‐Cs, and ChIA‐PETs. Those experiments, however, are much more expensive than the simple WGS. That is why, we believe that our findings show that with the advances in the machine learning field, we are closer and closer to decoding the DNA sequence—as we strongly believe, that everything is in the sequence, however strongly encoded, and not trivial to obtain. The modern NLP processing models are of a great help—as visible in our study, they can easily distinguish between the loops, whether it is mediated by CTCF or RNAPII, and the random parts of the genome. With the more advanced technology (as current SOTA tools, like DNABERT are still limited by the sequence length), we could create *in‐sillico* studies of millions of samples, without the need to conduct time‐consuming and expensive experiments, thus explaining the complex traits and diseases once and for all.

## MATERIALS AND METHODS

### Datasets

The proposed DHL approach is trained using ChIA‐PET interactions. The core idea of this study is to predict the CTCF & RNAPII ChIA‐PET interactions of length smaller than 1 Mb. This length was chosen as we are mostly interested in the interactions that happen within genetic domains, which have mean length of 1.15 Mb across multiple cell lines [22]. The chromatin interaction data is extracted from lymphoblastoid (GM12878) cell lines. The chromatin loops cause the parts of the genome far apart in 1D space to become spatially close, allowing them to interact. We define the short regions at the end of the loop as the anchors, which are considered to be CTCF/RNAPII binding sites.

An interaction is represented as two anchors, connecting distal parts of the genome. In the data preparation, each genomic subsequence of length 256 bp is retrieved from respective anchor regions considering the middle position of the anchor (±128 bp from the centre). Thus, the final data representation for a loop is a sequence of 512 bp separated by a delimiter token [SEP]. The negative set of interactions is generated by randomly selecting two genomic locations (excluding the positive data) following a similar positive data preparation strategy. The maximum interaction length (distance between two regions) is 1 Mb. This work considers 260,000 high‐quality CTCF and 160,000 high‐quality RNAPII interactions from the GM12878 cell line across all 23 chromosomes. The chromatin interactions from chromosome 9 are kept for validation, and the remaining interactions are used for training. The positive and negative sets are kept in a 1:1 ratio to achieve balanced learning.

### Methods

**Figure 2:**
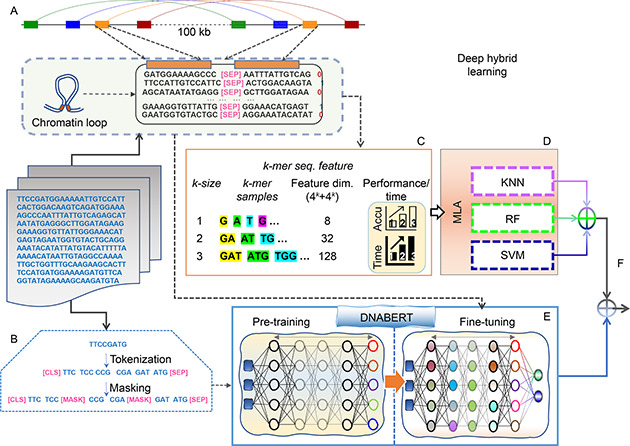
**Workflow of deep hybrid learning for chromatin loop prediction.** (A) Representation of subsequence pair separated by [SEP] token. (B) Tokenization strategy of sequences for BERT pretraining. (C) *k*‐mer feature representation of subsequence pair for machine learning classifiers, SVM, RF and KNN. (D) MLA model generation and classification. (E) Pretraining and fine‐tuning with labeled data with subsequence pairs and classification. (F) Hybridization of DNABERT and MLA based classification.

In this study, we have proposed a DHL algorithm to create a classifier for predicting chromatin loops incorporating transformer‐based deep learning (DNABERT) and three classical machine learning algorithms (RF, SVM, and KNN). The initial pre‐trained model originated from DNABERT [17] and was fine‐tuned to predict the loops. Fine‐tuning was done on the set, that was constructed as described in section of Datasets. Since we are dealing with long sequences, the chosen DNABERT pre‐trained model was *dnalongcat*. We have used learning rate of 2e−4 with warmup percent of 10%. We have also used 1% weight decay, and hidden dropout probability of 10%. All the remaining classical classifiers (used for the DHL) individually learn and build their models. In the primary step of the DHL, the logical operations are performed in two phases at the decision level: the first all three MLA classifiers are combined into a single decision; the second, this MLA‐based decision is ORed with DNABERT classification for final loop prediction. The detailed workflow of the methodology is depicted in the Fig.2 and logical formulation details are discussed in section of Deep hybrid learning.

#### Deep architecture: DNABERT

The Transformer‐based models are known for their exceptionally outstanding performance in various natural language processing (NLP) challenges. Their architecture includes an encoder and decoder and uses an entirely attention‐based approach. The DNABERT is a multilayer transformer encoder with two sub‐layers (multi‐head attention and fully connected feed‐forward layer). It uses a two‐step learning strategy. The first step, pre‐training, is used for learning the actual language, using as much unlabeled data as possible, thus learning connections between genetic sentences. In the fine‐tuning stage of learning, the system finds the solution for several task‐specific applications. DNABERT uses a set of nucleotide sequences—each represented as a set of *k‐*mers input tokens, and each token is embedded into a numeric vector. That *k*‐kmer approach helps connect single nucleotides together to show that they are not just random sequences—it adds the context of the single nucleotide by using its surroundings. The vocabulary of the DNA language is enriched with additional five tokens that are well‐known in the NLP tasks ([MASK] for masked tokens, [SEP] for separation tokens, [PAD] for padding tokens, [CLS] for classification tokens and [UNK] for unknown tokens) along with all permutations of *k‐*mers. Thus, in the vocabulary, there are $4^{k}+5$ unique tokens with 4 nucleotides (A, T, G, C) considering the token size as *k*. A masked token could be trivially predicted based on its previous and next *k‐*mers—that is why the masking strategy masks not only one token but also its surroundings—that way, the prediction is real, not just based on two closest neighbours. With the pre‐trained parameters of the DNABERT, we proposed incorporating the chromatin interaction prediction as the task‐specific fine‐tuning step. The metrics obtained using this approach for the validation set is shown in the Tab.1, and are significantly better than the ones obtained from strictly MLA approach.

#### Classical machine learning: support vector machine, random forest, K‐nearest neighbor classifiers

**Figure 3:**
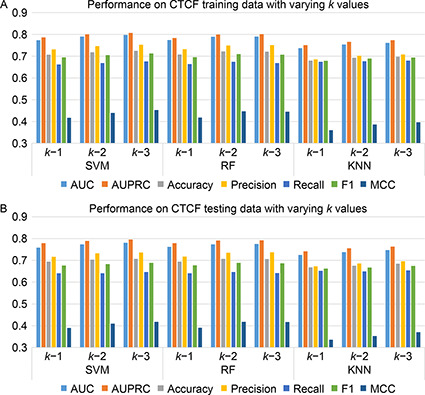
**The performance evaluation of CTCF data with varying *k* values on (A) training and (B) testing set** AUPRC, the area under the procision‐recall curve.

**Figure 4:**
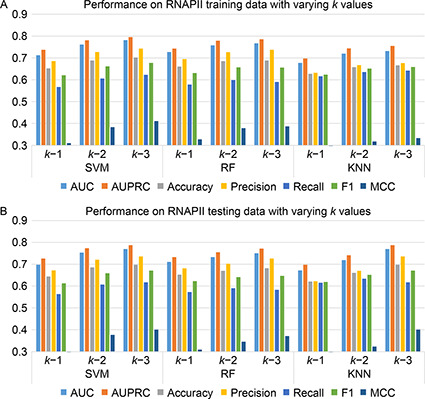
The performance evaluation of RNAPII data with varying *k* values on (A) training and (B) testing set.

We have incorporated three classical machine learning algorithms, SVM, RF, and KNN, for the chromatin loop prediction. The proposed method comprises *n*‐gram/ *k*‐mers feature representation from a pair of input sequences (one chromatin loop having two distant sequence regions). Initially, each sequence from a pair of input data is represented as a vector of all possible *k*‐mer frequencies and next, the vectors from both sequences are concatenated for the final feature representation [23]. We have checked the metrics using different values of *k* ranging *k*=1, 2, 3 and found that the performance for all three ML classifiers has significantly improved with the increasing value of *k*. However, increasing *k* values is computationally expensive, and the processing time increases exponentially. Thus the *k* value is set to 3 in this experiment for final performance evaliation. The results for all three ML classifiers with different *k* values in the CTCF and RNAPII data set are shown in the Fig.3 and Fig.4, respectively.

**Figure 5:**
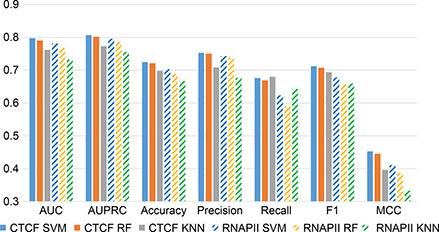
The performance evaluation of the CTCF and RNAPII dataset in five‐fold cross‐validation.

Finally, we set the *k*‐value as 3 for feature generation purposes in all MLA learning. It results in 128 ($4^{3}+4^{3}$ ) dimensional features for the MLA algorithms. The models from all MLA classifiers are evaluated individually with a 5‐fold cross‐validation scheme. Finally, the best models are selected to contribute to deep hybrid learning. The fold‐wise learning performances have been reported in the Fig.5.

#### Deep hybrid learning

In our study, we are utilising the power of a deep hybrid learning model that combines the attention‐based transformer learning in BERT architecture derivative (DNABERT) with a classical machine learning approach (SVM, RF, KNN) for chromatin loop prediction. In this study, the hybrid strategy has shown potential for reducing the prediction error in the task of chromatin interaction prediction. Initially, all three classifiers are trained with the training dataset independently. The best models from each classical machine learning algorithm are combined with the DNABERT results. In the first phase of the DHL, all MLAs are integrated into a single decision using logical operation between the individual class labels and their majority voting count. Let a given set of three Boolean literals $\left\{l_{s},l_{r},l_{k}\right\}$ represent the predicted class labels of three MLA models for any datapoint t . Now, we can define the majority voting based decision $l_{mv}$ as a Boolean operation.

$$l_{mv}=\left\{ \begin{array}{c} 1,\;if\;sum\left(l_{s},l_{r},l_{k}\right)\geq 2\\ 0,\;otherwise \end{array}\right.$$

Final decision for the data point t from all three classifiers is defined as, $f\left(t\right)=l_{mv}⋏{(l}_{s}⋎l_{r}⋎l_{k})$ , where ⋏ and ⋎ represent the logical AND and OR operation respectively. At the final stage, DNABERT results are combined with the MLAs’ decisions (f(t) ) with logical OR operation for final classification.

## SUPPLEMENTARY MATERIALS

The description of all the detailed fold‐wise experiment and individual fold‐specific resuls and hold‐out result tables are listed as Tables. S1−S12, and can be found online with this article at https://doi.org/10.15302/J‐QB‐022‐0315.

## COMPLIANCE WITH ETHICS GUIDELINES

The authors Mateusz Chiliński, Anup Kumar Halder and Dariusz Plewczynski declare that they have no conflict of interest or financial conflicts to disclose.

All procedures performed in studies involving animals were in accordance with the ethical standards of the institution or practice at which the studies were conducted, and with the 1964 Helsinki declaration and its later amendments or comparable ethical standards.

## Supporting information

Supplementary Information
