# Supplementary material for: Prediction of chromatin looping using deep hybrid learning (DHL)
Source: Quant Biol. 2023 Jun 1;11(2):155–62. doi: 10.15302/J-QB-022-0315 (PMC12806927; doi:10.15302/J-QB-022-0315)
Supplement: Supplementary file 1 — Supplementary Information [file QUB2-11-155-s001.pdf]

CTCF

Result: FOLD-WISE (Test Data)

Tab. S1. Foldwise performance evaluation on the test data (one-versus-all) with all three classifiers considering k=3

KMR=3

Classifier	Fold	AUC	AUPRC	Accurac	Precisio	Recall	F1	MCC
SVM	0	0.8	0.8051	0.7255	0.7498	0.6831	0.7149	0.4532
	1	0.802	0.8068	0.7343	0.7585	0.686	0.7204	0.4707
	2	0.79	0.8033	0.7187	0.7502	0.6635	0.7042	0.4409
	3	0.794	0.8052	0.7207	0.7535	0.6684	0.7084	0.4451
	4	0.803	0.8137	0.7258	0.7507	0.6792	0.7132	0.4539
	**Avg**	**0.798**	**0.8068**	**0.725**	**0.7525**	**0.676**	**0.7122**	**0.4528**

Classifier	Fold	AUC	AUPRC	Accuracy	Precision	Recall	F1	MCC
RF	0	0.793	0.8012	0.7287	0.7567	0.6801	0.7164	0.4601
	1	0.792	0.7996	0.7272	0.7488	0.682	0.7139	0.456
	2	0.784	0.7991	0.7168	0.7473	0.6631	0.7027	0.437
	3	0.787	0.7973	0.7147	0.7528	0.652	0.6988	0.4344
	4	0.795	0.8107	0.7192	0.7468	0.6662	0.7042	0.4411
	**Avg**	**0.79**	**0.8016**	**0.7213**	**0.7505**	**0.6687**	**0.7072**	**0.4457**

Classifier	Fold	AUC	AUPRC	Accuracy	Precision	Recall	F1	MCC
KNN	0	0.765	0.7728	0.7007	0.7095	0.6874	0.6983	0.4016
	1	0.768	0.7781	0.7057	0.7091	0.6954	0.7022	0.4114
	2	0.753	0.7628	0.6903	0.7035	0.6678	0.6852	0.3814
	3	0.757	0.7779	0.6905	0.7079	0.6645	0.6855	0.3822
	4	0.765	0.7741	0.7027	0.7121	0.6842	0.6978	0.4057
	**Avg**	**0.762**	**0.7731**	**0.698**	**0.7084**	**0.6799**	**0.6938**	**0.3965**

Tab. S3. Foldwise performance evaluation on the test data (one-versus-all) with all three classifiers considering k=2

Classifier	Fold	AUC	AUPRC	Accuracy	Precision	Recall	F1	MCC
SVM	0	0.793	0.7979	0.7207	0.7497	0.6689	0.707	0.4444
	1	0.795	0.8007	0.7268	0.7501	0.6787	0.7126	0.4556
	2	0.782	0.797	0.7118	0.7403	0.6608	0.6983	0.4267
	3	0.786	0.7997	0.7125	0.7456	0.6582	0.6992	0.429
	4	0.796	0.8079	0.7202	0.7438	0.6749	0.7076	0.4424
	**Avg**	**0.79**	**0.8006**	**0.7184**	**0.7459**	**0.6683**	**0.7049**	**0.4396**

Classifier	Fold	AUC	AUPRC	Accuracy	Precision	Recall	F1	MCC
	0	0.791	0.7987	0.7247	0.7488	0.6824	0.7141	0.4515

KMR=2

RF	1	0.792	0.7968	0.7278	0.7486	0.6844	0.7151	0.4573
	2	0.783	0.7974	0.7177	0.7467	0.6668	0.7045	0.4384
	3	0.785	0.7944	0.7162	0.7518	0.6582	0.7019	0.4368
	4	0.795	0.8089	0.7255	0.7511	0.6775	0.7124	0.4534
	**Avg**	**0.789**	**0.7992**	**0.7224**	**0.7494**	**0.6739**	**0.7096**	**0.4475**

Classifier	Fold	AUC	AUPRC	Accuracy	Precision	Recall	F1	MCC
KNN	0	0.755	0.7669	0.6998	0.7087	0.6864	0.6974	0.4
	1	0.759	0.7671	0.6987	0.7013	0.69	0.6956	0.3974
	2	0.755	0.7684	0.698	0.7135	0.6711	0.6916	0.397
	3	0.745	0.7607	0.6773	0.6906	0.6602	0.6751	0.3553
	4	0.755	0.7668	0.691	0.6986	0.6759	0.687	0.3823
	**Avg**	**0.754**	**0.766**	**0.693**	**0.7025**	**0.6767**	**0.6893**	**0.3864**

Tab. S5. Foldwise performance evaluation on the test data (one-versus-all) with all three classifiers considering k=1

KMR=1

Classifier	Fold	AUC	AUPRC	Accuracy	Precision	Recall	F1	MCC
SVM	0	0.776	0.7843	0.7102	0.7314	0.6712	0.7	0.4221
	1	0.778	0.7867	0.7117	0.7293	0.6713	0.6991	0.4246
	2	0.766	0.7842	0.7053	0.7332	0.6542	0.6914	0.4137
	3	0.77	0.7844	0.703	0.7332	0.6523	0.6904	0.4094
	4	0.778	0.7941	0.707	0.7289	0.6626	0.6942	0.4159
	**Avg**	**0.774**	**0.7867**	**0.7074**	**0.7312**	**0.6623**	**0.695**	**0.4171**

Classifier	Fold	AUC	AUPRC	Accuracy	Precision	Recall	F1	MCC
RF	0	0.777	0.7857	0.7107	0.7324	0.6709	0.7003	0.4232
	1	0.778	0.7842	0.7132	0.7305	0.6737	0.701	0.4275
	2	0.769	0.7803	0.7022	0.7296	0.6513	0.6882	0.4073
	3	0.77	0.7809	0.7052	0.7359	0.654	0.6925	0.4139
	4	0.779	0.7896	0.7102	0.7313	0.6679	0.6981	0.4221
	**Avg**	**0.775**	**0.7841**	**0.7083**	**0.7319**	**0.6636**	**0.696**	**0.4188**

Classifier	Fold	AUC	AUPRC	Accuracy	Precision	Recall	F1	MCC
KNN	0	0.735	0.751	0.6762	0.6827	0.6675	0.675	0.3525
	1	0.746	0.7574	0.6852	0.6835	0.6874	0.6854	0.3703
	2	0.732	0.7453	0.6792	0.6859	0.6721	0.6789	0.3585
	3	0.73	0.7482	0.6763	0.6887	0.6615	0.6748	0.3532
	4	0.741	0.7548	0.683	0.6854	0.6808	0.6831	0.366
	**Avg**	**0.737**	**0.7513**	**0.68**	**0.6852**	**0.6739**	**0.6794**	**0.3601**

Result: FOLD-WISE (HoldOut Data)

Tab. S2. Foldwise performance evaluation on holdout test with all three classifiers considering k=3

Classifier	Fold	AUC	AUPRC	Accurac	Precisio	Recall	F1	MCC
SVM	0	0.7814	0.7959	0.7076	0.7361	0.6472	0.6888	0.4183
	1	0.7811	0.7958	0.7074	0.736	0.6468	0.6885	0.4179
	2	0.7808	0.7956	0.7074	0.7348	0.649	0.6893	0.4177
	3	0.781	0.7956	0.7074	0.7364	0.6459	0.6882	0.4179
	4	0.7814	0.796	0.7076	0.7363	0.6468	0.6887	0.4183
	**Avg**	**0.7811**	**0.7958**	**0.7075**	**0.7359**	**0.6471**	**0.6887**	**0.418**
	**WD**	0.7826	0.7968	0.7087	0.7369	0.6491	0.6902	0.4203

Classifier	Fold	AUC	AUPRC	Accuracy	Precision	Recall	F1	MCC
RF	0	0.7752	0.7919	0.7067	0.7379	0.6412	0.6861	0.417
	1	0.7756	0.7925	0.7075	0.7369	0.6453	0.6881	0.4181
	2	0.7749	0.7916	0.7059	0.7355	0.6432	0.6863	0.4152
	3	0.7749	0.7917	0.7069	0.7404	0.6374	0.685	0.418
	4	0.7759	0.7926	0.7073	0.7378	0.6431	0.6872	0.418
	**Avg**	**0.7753**	**0.7921**	**0.7069**	**0.7377**	**0.642**	**0.6865**	**0.4173**
	**WD**	0.7744	0.7916	0.7057	0.7355	0.6425	0.6859	0.4148

Classifier	Fold	AUC	AUPRC	Accuracy	Precision	Recall	F1	MCC
KNN	0	0.748	0.7624	0.6856	0.6964	0.6582	0.6768	0.3719
	1	0.7466	0.7635	0.6832	0.6925	0.6588	0.6752	0.3667
	2	0.7471	0.7643	0.6828	0.6951	0.6513	0.6725	0.3663
	3	0.748	0.7647	0.6881	0.7027	0.6522	0.6765	0.3772
	4	0.7456	0.7614	0.6833	0.6952	0.6527	0.6733	0.3672
	**Avg**	**0.7471**	**0.7633**	**0.6846**	**0.6964**	**0.6546**	**0.6749**	**0.3699**
	**WD**	0.7485	0.7656	0.6846	0.6976	0.6518	0.6739	0.3701

Tab. S4. Foldwise performance evaluation on holdout test with all three classifiers considering k=2

Classifier	Fold	AUC	AUPRC	Accuracy	Precision	Recall	F1	MCC
SVM	0	0.7742	0.7896	0.7037	0.734	0.6392	0.6833	0.4109
	1	0.774	0.7895	0.7023	0.7297	0.6425	0.6833	0.4074
	2	0.7741	0.7897	0.7035	0.7319	0.6422	0.6841	0.4101
	3	0.7738	0.7893	0.7036	0.7332	0.6401	0.6835	0.4105
	4	0.7742	0.7896	0.7029	0.7322	0.6399	0.683	0.4092
	**Avg**	**0.7741**	**0.7895**	**0.7032**	**0.7322**	**0.6408**	**0.6834**	**0.4096**

Classifier	Fold	AUC	AUPRC	Accuracy	Precision	Recall	F1	MCC
	0	0.7731	0.7911	0.7069	0.7343	0.6484	0.6887	0.4167

**RF**	1	0.7742	0.7913	0.7077	0.735	0.6496	0.6897	0.4182
	2	0.7732	0.7907	0.7077	0.7348	0.6502	0.6899	0.4183
	3	0.7736	0.791	0.7076	0.7383	0.643	0.6874	0.4186
	4	0.7738	0.7913	0.7067	0.7365	0.6436	0.6869	0.4166
	**Avg**	**0.7736**	**0.7911**	**0.7073**	**0.7358**	**0.647**	**0.6885**	**0.4177**

**Classifier**	Fold	AUC	AUPRC	Accuracy	Precision	Recall	F1	MCC
**KNN**	0	0.7361	0.755	0.6738	0.6849	0.6439	0.6637	0.3482
	1	0.7391	0.7569	0.6756	0.6836	0.654	0.6685	0.3516
	2	0.7387	0.7555	0.6753	0.687	0.644	0.6648	0.3513
	3	0.7385	0.7552	0.678	0.6873	0.6531	0.6697	0.3563
	4	0.7384	0.7556	0.6772	0.6861	0.6534	0.6693	0.3548
	**Avg**	**0.7382**	**0.7556**	**0.676**	**0.6858**	**0.6497**	**0.6672**	**0.3524**

Tab. S6. Foldwise performance evaluation on holdout test with all three classifiers considering k=1

**Classifier**	Fold	AUC	AUPRC	Accuracy	Precision	Recall	F1	MCC
**SVM**	0	0.7589	0.7795	0.6938	0.7166	0.6411	0.6768	0.3898
	1	0.7589	0.7795	0.6938	0.7165	0.6415	0.6769	0.3898
	2	0.759	0.7796	0.6943	0.7175	0.6407	0.677	0.3907
	3	0.7589	0.7795	0.6944	0.7177	0.6408	0.6771	0.3911
	4	0.7591	0.7797	0.6933	0.7163	0.6401	0.6761	0.3888
	**Avg**	**0.759**	**0.7796**	**0.6939**	**0.7169**	**0.6408**	**0.6768**	**0.39**

**Classifier**	Fold	AUC	AUPRC	Accuracy	Precision	Recall	F1	MCC
**RF**	0	0.762	0.7795	0.6944	0.7173	0.6416	0.6773	0.3909
	1	0.7624	0.779	0.695	0.7178	0.6428	0.6782	0.3922
	2	0.7621	0.7794	0.6946	0.7174	0.6419	0.6776	0.3913
	3	0.762	0.7791	0.6935	0.717	0.6396	0.6761	0.3894
	4	0.7625	0.7792	0.6948	0.7187	0.6402	0.6772	0.3919
	**Avg**	**0.7622**	**0.7792**	**0.6945**	**0.7176**	**0.6412**	**0.6773**	**0.3911**

**Classifier**	Fold	AUC	AUPRC	Accuracy	Precision	Recall	F1	MCC
**KNN**	0	0.7249	0.7432	0.6674	0.672	0.6543	0.663	0.335
	1	0.7255	0.7423	0.6674	0.6726	0.6524	0.6624	0.3351
	2	0.7266	0.7418	0.6693	0.6758	0.6508	0.6631	0.3388
	3	0.7233	0.7414	0.6658	0.6719	0.648	0.6597	0.3318
	4	0.7247	0.7398	0.6685	0.6732	0.6549	0.6639	0.3371
	**Avg**	**0.725**	**0.7417**	**0.6677**	**0.6731**	**0.6521**	**0.6624**	**0.3356**
